# Moderate Dietary Protein Restriction Optimized Gut Microbiota and Mucosal Barrier in Growing Pig Model

**DOI:** 10.3389/fcimb.2018.00246

**Published:** 2018-07-18

**Authors:** Xiyue Chen, Peixia Song, Peixin Fan, Ting He, Devin Jacobs, Crystal L. Levesque, Lee J. Johnston, Linbao Ji, Ning Ma, Yiqiang Chen, Jie Zhang, Jinshan Zhao, Xi Ma

**Affiliations:** ^1^State Key Laboratory of Animal Nutrition, College of Animal Science and Technology, China Agricultural University, Beijing, China; ^2^Department of Animal Sciences, South Dakota State University, Brookings, SD, United States; ^3^Swine Nutrition and Production, West Central Research and Outreach Center, University of Minnesota, Morris, MN, United States; ^4^Department of Animal Husbandry and Veterinary, Beijing Vocational College of Agriculture, Beijing, China; ^5^Key Laboratory of Animal Nutrition, College of Animal Science and Technology, Qingdao Agricultural University, Qingdao, China; ^6^Department of Internal Medicine, Department of Biochemistry, University of Texas Southwestern Medical Center, Dallas, TX, United States

**Keywords:** dietary protein restriction, growing pigs, intestinal microbiota, mucosal barrier, intestinal stem cell

## Abstract

Appropriate protein concentration is essential for animal at certain stage. This study evaluated the effects of different percentages of dietary protein restriction on intestinal health of growing pigs. Eighteen barrows were randomly assigned to a normal (18%), low (15%), and extremely low (12%) dietary protein concentration group for 30 days. Intestinal morphology and permeability, bacterial communities, expressions, and distributions of intestinal tight junction proteins, expressions of biomarkers of intestinal stem cells (ISCs) and chymous bacterial metabolites in ileum and colon were detected. The richness and diversity of bacterial community analysis with Chao and Shannon index were highest in the ileum of the 15% crude protein (CP) group. Ileal abundances of *Streptococcaceae* and *Enterobacteriaceae* decreased respectively, while beneficial *Lactobacillaceae, Clostridiaceae*_*1, Actinomycetaceae*, and *Micrococcaceae* increased their proportions with a protein reduction of 3 percentage points. Colonic abundances of *Ruminococcaceae, Christensenellaceae, Clostridiaceae*_*1, Spirochaetaceae*, and *Bacterodales_S24-7_group* declined respectively, while proportions of *Lachnospiraceae, Prevotellaceae*, and *Veillonellaceae* increased with dietary protein reduction. Concentrations of most bacterial metabolites decreased with decreasing dietary protein concentration. Ileal barrier function reflected by expressions of tight junction proteins (occludin, zo-3, claudin-3, and claudin-7) did not show significant decrease in the 15% CP group while sharply reduced in the 12% CP group compared to that in the 18% CP group. And in the 15% CP group, ileal distribution of claudin-3 mainly located in the cell membrane with complete morphological structure. In low-protein treatments, developments of intestinal villi and crypts were insufficient. The intestinal permeability reflected by serous lipopolysaccharide (LPS) kept stable in the 15% CP group while increased significantly in the 12% CP group. The expression of ISCs marked by Lgr5 slightly increased in ileum of the 15% CP group. Colonic expressions of tight junction proteins declined in extremely low protein levels. In conclusion, moderate protein restriction (15% CP) can optimize the ileal microbiota structure via strengthening beneficial microbial populations and suppressing harmful bacterial growth and altering the function of ileal tight junction proteins as well as epithelial cell proliferation.

## Introduction

Dietary protein is a fundamental nutrient for animals and essential for organ physiological function. The shortage of protein sources and environmental pollution caused by large-scale nitrogen emissions have become bottlenecks restricting the sustainable development of pig husbandry. The dietary crude protein (CP) level recommended for growing pigs (20–50 kg) is 18% according to National Research Council (2012). Low-protein diets have received extensive attention in recent years due to their advantages in saving protein resources and reducing nitrogen emissions. Several researchers have reported that restricting dietary protein level by 3 percentage points could promote lipid and energy metabolism of skeletal muscle in growing pigs without affecting growth performance, while protein reduction of more than 4 percentage points would reduce growth performance (Wood et al., 2013; Herrero et al., 2016). There is ample evidence that reducing dietary intake of protein and simultaneously supplementing crystalline amino acids enable the improvement of gut health and optimization of nitrogen utilization for protein accretion (Zhou et al., 2016).

The integrity of the intestinal structure and dynamic balance of intestinal microbiota guarantee the chemical induction and digestive functions of the gut, which is the premise for nutrient absorption, metabolism, and deposition. The intestinal microbiota, acknowledged as a crucial factor in maintaining intestinal functions (Sczesnak et al., 2011; Chen et al., 2015), is a complex ecosystem with almost 100 trillion microorganisms, most of which are bacteria (Collins et al., 2012).

There are many factors influencing the composition and activity of gut microbiota including diet, environment, and age; among them diet is the most important (Fan et al., 2015; Sonnenburg et al., 2016; Ma N. et al., 2017). Diet plays an essential role in shaping the composition of the gut microbiota and the status of immune response modulated by gut microbiota (Saresella et al., 2017). Specifically, for growing pigs, proteins function as the building blocks for tissues. Moderate dietary protein restriction could alter the composition of gut microbiota and improve ileal barrier function in adult pigs (Fan et al., 2017a). The microbial composition, along with a wide range of microbial metabolites, plays a complex role in various host processes, such as energy harvest, recovery from inflammation and infection, resistance to autoimmunity, and endocrine signaling that affects brain function through the gut-brain axis (Hooper et al., 2012; Hollister et al., 2014; Han et al., 2016; Chen et al., 2017; He et al., 2017). Small-intestinal or colonic microbiota have also been considered as potential amino acid sources in animals (Miller and Ullrey, 1987).

Growing pigs have been proposed as an alternative animal model for children as they have similar anatomical and physiological characteristics (Miller and Ullrey, 1987). Protein restriction has been proved to modulate the growth of muscle fiber in growing-finishing pigs, while limited attention has been given to its regulation of microbiota populations. Herein, growing pigs were selected as the experimental model, and the effects of decreasing dietary protein concentration on gut health in terms of gut microbiota, intestinal barrier function, and proliferation of intestinal stem cells (ISCs) were investigated.

## Materials and methods

### Ethics

All management and experimental procedures followed the animal care protocols approved by the China Agricultural University Animal Care and Use Ethics Committee (CAU20160928-2). All experimental protocols were approved and the methods were conducted according to the relevant guidelines and regulations.

### Animals, diet treatments, and sampling

A total of 18 barrows (Duroc × Landrace × Yorkshire) with initial body weight (BW) 36.52 ± 0.49 kg were selected from 4 litters. These pigs were randomly assigned to 3 treatments with 6 pigs per treatment on the basis of similar BW. Pigs were fed three diets including a normal dietary protein concentration (18%), low dietary protein concentration (15%), and extremely low dietary protein concentration (12%). The basal diet was formulated to satisfy nutrient specifications (National Research Council, 2012) for 20–50 kg BW pigs (Supplemental Table 1). Formulated diets contained similar digestible energy (DE) content and standardized ileal digestible (SID) contents of essential amino acids including lysine, methionine plus cysteine, tryptophan and threonine. All pigs were housed with one pig per pen and fed *ad libitum* throughout the entire experiment. All pigs were slaughtered 30 days later. The chyme and intestinal segments in the middle part of ileum and colon, as well as serum were collected.

### DNA extraction and PCR amplification

DNA extraction of intestinal contents was conducted using the DNA Stool Mini Kit (Qiagen, Hilden, Germany) following manufacturer's protocols. The bacterial universal V3–V4 region of the 16S rRNA gene was amplified using PCR bar-coded primers 338F (5′-ACTCCTACGGGAGGCAGCA-3′) and 806R (5′-GGACTACHVGGGTWTCTAAT-3′). PCR was set in 20 μL volume, with 1 × FastPfu buffer, 250 μM dNTP, 1 U FastPfu polymerase, 0.1 μM each of the primer and 10 ng template DNA. PCR was conducted at 95°C for 2 min and 30 cycles of 95°C for 30 s, then annealed at 55°C for 30 s, 72°C for 30 s, and extended at 72°C for 5 min.

### Illumina sequencing and data analysis

Amplicons were detected with 2% agarose gel electrophoresis and purified with the AxyPrep DNA Purification Kit (Axygen Biosciences, Union City, CA, USA). PCR products were then visualized on agarose gels and were determined quantitatively with PicoGreen dsDNA Quantitation Reagent (Invitrogen, Carlsbad, USA) and QuantiFluor-ST Fluoremeter (Promega, USA). Purified amplicons were pooled with an Illumina MiSeq platform (Majorbio, Shanghai, China) following the standard protocols in equimolar and paired-end sequenced (2 × 300). The raw data were uploaded to NCBI SRA Database, with the SRA accession: SRP111417.

### Bacterial data processing

Sequencing data were subjected to bioinformatics analysis. QIIME (version 1.17) was used to demultiplex and quality-filter raw FASTQ files with the following criteria: (i) the 300 bp reads at any site obtaining an average quality score of < 20 over a 10 bp sliding window were cut, with reads shorter than 50 bp abandoned; (ii) exact match of barcode, with 2 nucleotide mismatching in primer match, or reads containing ambiguous characters were removed; (iii) according to their overlapped sequence, overlapping sequences were selected with the minimum length of 10 bp. Reads that could not be assembled were abandoned. The sequences were clustered into operational taxonomic units (OTUs) with UPARSE (version 7.1: http://drive5.com/uparse/) with a novel “greedy” algorithm that performs chimera filtering and OTU clustering simultaneously and the identity threshold was set at 97%. OTUs with only one sequence were removed and UCHIME was used to identify and remove chimeric sequences (Hao et al., 2017). The rarefaction analysis with Mothur v.1.21.1 was performed to reflect the diversity indices. The software Primer 6 (Primer-E Ltd., UK) was used for hierarchical clustering analysis. R tools were used to generate community figures with the data from document “tax.phylum.xls, tax.family.xls, and tax. genus.xls.” The bar figures of bacterial community were conducted with R ggplot package and heatmaps were conducted with R vegan package. For statistical analysis at the genus level, R stats and scipy of python packages were used.

### Detection of short-chain fatty acids (SCFAs) in ileal and colonic contents by gas chromatography

The concentrations of SCFAs were detected using a gas chromatographic method according to the protocols from a previous report (Singh et al., 2014) with a few changes. In brief, 1.5 g ileal or colonic digesta was suspended in 1.5 mL distilled water and centrifuged at 15,000 × g at 4°C for 10 min. One milliliter of supernatant mixed with 200 μL of metaphosphoric acid was put in an ampoule and set in an ice bath for 30 min and centrifuged for 10 min. Samples were inserted into a HP 6890 Series Gas Chromatograph (Hewlett Packard, PA, California) with a HP 19091N-213 column with 30.0 m × 0.32 mm i.d. (Agilent, PA, California). Temperatures for injector and detector were set at 185°C and 210°C, respectively. Each sample was measured three times.

### Determination of biogenic amines in ileal and colonic digesta with high performance liquid chromatography (HPLC)

The concentrations of methylamine, spermidine, cadaverine, putrescine, and histamine in chyme were detected by HPLC with a method adapted from the previous report (Fan et al., 2017b) with modifications. Briefly, 0.2 g ileal or colonic chyme was put into a 2 mL screw-capped tube along with 1 mL trichloroacetic acid solution, then homogenized for 10 min and centrifuged at 3,600 × g at 4°C for 10 min. An equal volume of n-hexane was mixed with pooled supernatant for 5 min. The organic phase was abandoned and the same protocols were used to re-extract the water phase. The pretreated sample was mixed with 20 mL internal standard saturated sodium bicarbonate solution, 1 mL dansyl chloride, and 1 mL NaOH, then set at 60°C for 45 min with several soft reversing. The reaction was then stopped by adding 100 μL ammonia. The solution was maintained in the 40°C water bath and the acetone was vaporized with nitrogen flow. The solution was finally extracted twice with diethyl ether. The extract was dried with nitrogen flow and the residue for injection was dissolved in acetonitrile. HPLC analysis was performed with elution of ammonium acetate-acetonitrile gradient and an Agilent 1200 series system with a dual low-pressure gradient pump, as well as an auto sampler and a column compartment and variable wavelength detector (VWD). The column was a reversed-phase ZORBAX 80A Extend-C18 (4.6 mm × 250 mm; 5 μm) (Agilent, Santa Clara, CA, USA). The two solvent reservoirs contained acetonitrile and 0.2 mol/L ammonium acetate. The flow rate was 1.0 mL/min and the temperature was 30°C. The wavelength was 260 nm for VWD. Each sample was measured three times.

### Intestinal morphology and permeability

Samples of ileal and colonic tissues from each growing pig were fixed immediately in polyformaldehyde for intestinal morphological observation. The tissues were then dehydrated and embedded. The tissues, in paraffin blocks, were cut to 4 μm sections, and stained with haematoxylin and eosin. Samples were measured with a BX-51 microscope (Olympus). Representative photographs of the intestinal morphology of the ileum and colon were recorded with Visitron Systems (Puchheim). NIS-Elements BR Software (Nikon, Version 2.20) was used to measure the villi height, crypt depth and area of colonic epithelial cells. Intestinal permeability was detected with serous lipopolysaccharide (LPS) using assay kits according to the manufacturer's instructions (Jonln, Jiangsu, China).

### Extraction of protein and immunoblotting

Total protein was extracted from intestinal tissues with lysis buffer (150 mM NaCl, 1% Triton X-100, 50 mM Tris-HCl, 0.5% sodium deoxycholate, 0.1% SDS at pH 7.4, adding a protease inhibitor (Applygene, Beijing, China). A sample (0.02 g) of each frozen intestinal segment was powdered with liquid nitrogen. Lysis buffer with protease inhibitors was used for lysis and products were centrifuged at 10,000 × g for 5 min at 4°C and the supernatant were harvested. A BCA Protein Assay Kit (Pierce, Rockford, IL) was used to determine total protein concentrations. Proteins of equal amounts (40 μg) were electrophoresed with SDS polyacrylamide gel. Proteins were transferred to a nitrocellulose membrane (Millipore, Bedford, MA, USA) electrophoretically, blocked with Tris-buffered saline with 0.05% Tween-20 (TBS-T) and 5% non-fat milk (at room temperature for 1 h), then incubated with primary antibodies against claudin-1, leucine-rich repeat-containing G-protein-coupled receptor 5 (Lgr5) (Santa Cruz Biotechnology, Santa Cruz, CA), occludin, claudin-3, claudin-7, zonula occludens protein 3 (ZO-3), bmi1 polycomb ring finger oncogene (Bmi1) (Abcam, Cambridge, United Kingdom), and β-actin (Cell Signalling Technology, Danvers, MA, USA) overnight at 4°C. Membranes were washed with TBS-T three times and incubated with the appropriate secondary antibodies in the dark (at room temperature for 2 h). The membranes were washed three times with TBS-T. Signals were detected and quantified with LI-COR Infrared Imaging System (Odyssey, Lincoln, NE).

### Immunofluorescence assay

The pretreatment of ileal and colonic samples were the same as the detection of intestinal morphology and the following procedure referred to a previous report (Ma X. et al., 2017). The 4 μm sections were dewaxed twice with xylene for 10 min and rehydrated with alcohol. Ten minute of 0.5% Triton X-100 was used for permeabilization. Antigen retrieval was conducted with sodium citrate for 20 min. After washing with PBS for 3 times, the samples were rinsed in H_2_O_2_ for 10 min and then blocked with 10% serum for 1 h. For Claudin-3 staining, ileal, and colonic sections were incubated with claudin-3 antibody (Abcam) overnight at 4°C. After washing with PBS 3 times, the sections were incubated with secondary antibodies in the dark at 37°C for 1 h and DAPI (Invitrogen, California) for 10 min. Staining was detected using a laser scanning microscope (Zeiss LSM 510 META Nlo).

### Statistical analysis

Data were analyzed with STAMP and SAS Version 9.2. Fisher' exact test was used for bacterial data (Parks and Beiko, 2010). Other data were analyzed with simple one-way ANOVA with Duncan's multiple range test. Differences were considered significant when *P* < 0.05.

## Results

### Intestinal bacterial richness, diversity, and similarity

**Figure 1:**
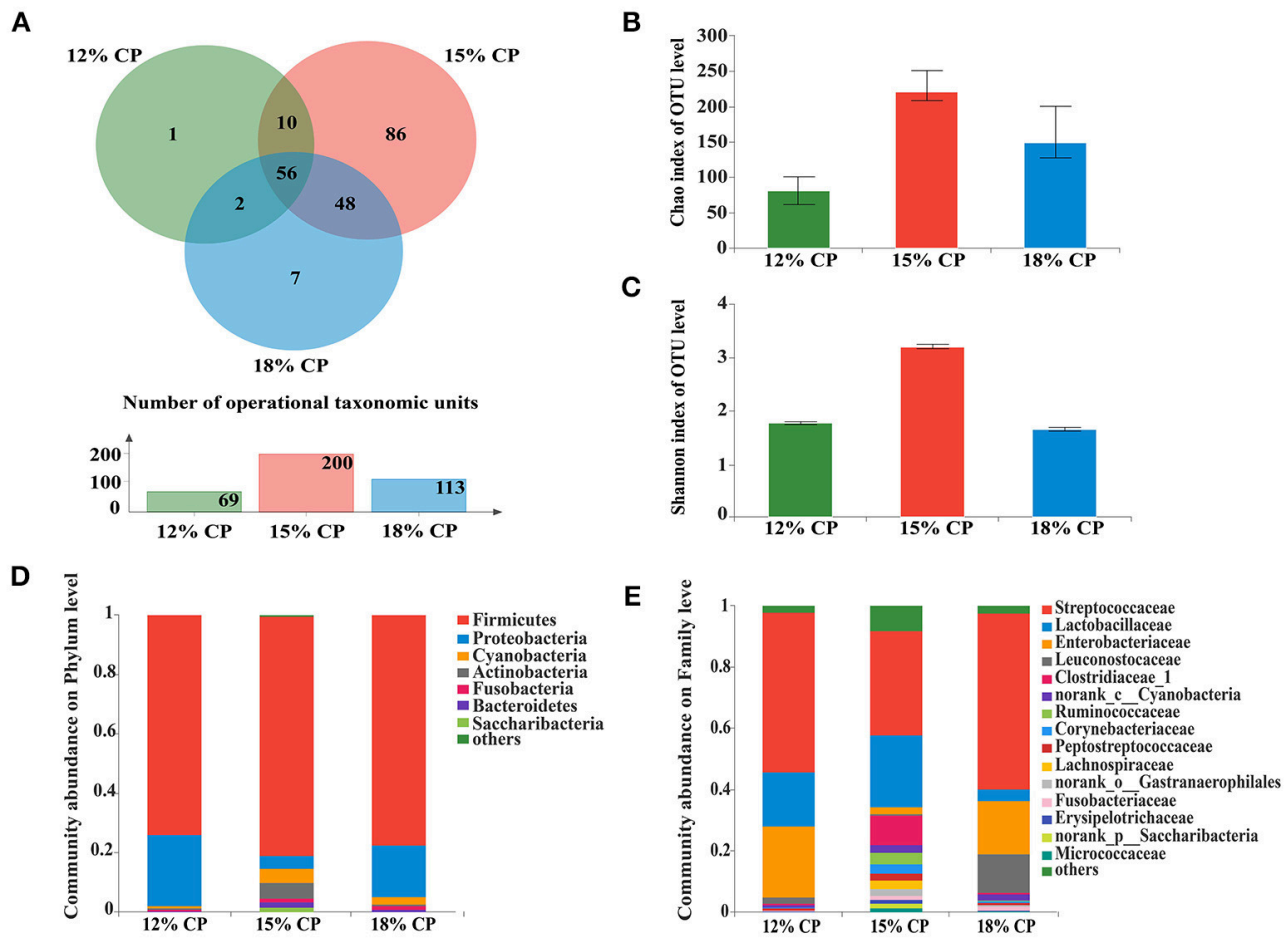
Effects of low-protein diets on the richness and composition of ileal microbiota in growing pigs at the phylum and family levels. **(A)** Venn of the operational taxonomic units (OTUs) among different dietary treatments in the ileum. **(B)** Chao index of the bacterial community in the ileum. **(C)** Shannon index of the bacterial community in the ileum. **(D)** Distribution of ileal bacteria at the phylum level with relative abundance higher than 1%. **(E)** Distribution of ileal bacteria at the family level with relative abundance higher than 1%.

Valid sequences (*n* = 83,536) were obtained after size filtering, quality control and chimera removal, with an average of 12,077 ± 3,521 sequences per ileal sample and an average of 15,768 ± 896 sequences per colonic sample. At the distance level of 0.03 (97% similarity), 210 distinct OTU were detected in ileal samples (Figures 1A, 3A) and 304 in colonic samples (Figure 3A), of which 56 shared bacteria in the ileum and 174 in the colon were represented among the 3 diet groups. The colonic bacterial community (Figures 3B,C) had higher Chao estimate and Shannon index than the ileal bacterial community (Figures 1B,C).

When the concentration of dietary protein reached 15%, the Chao estimate and Shannon index of the bacterial community tended to be highest in the ileum (Figures 1B,C). This indicated that the bacterial community in the ileum of the 15% CP group had the highest diversity and richness. However, the lowest Chao index and Shannon index were observed in the colons of the 15% CP group and 18% CP group, respectively (Figures 3B,C).

### Ileal bacterial community structure

Ileal bacterial community of three groups shared 27% OTUs according to the Venn diagram (Figure 1A). Different groups had diverse amounts of unique OTUs, with 15% CP group being the most and 12% CP group being the least, indicating that dietary protein level significantly affected ileal bacterial community structure.

*Firmicutes, Proteobacteria, Cyanobacteria*, and *Actinobacteria* were the four major bacterial phyla in ileal contents of growing pigs, accounting for more than 90% of the total ileal bacterial community (Figure 1D); however, their respective proportion varied with the reduction of dietary protein concentration. When protein concentration was reduced from 18% to 15% and 12%, abundance of *Firmicutes* increased from 73.88% to 81.22% (*P* < 0.05) and then decreased to 77.46% (*P* < 0.05), respectively, while abundance of *Proteobacteria* varied from 24.02% to 4.18% (*P* < 0.05) and 17.72% (*P* < 0.05), respectively. Proportion of *Cyanobacteria* and *Actinobacteria* in the 15% CP group held at 4.81% and 5.33%, respectively, which were notably higher than the other two groups (*P* < 0.05). In sum, dietary protein concentration of 15% could apparently alter ileal bacterial community structure.

At the family level, *Streptococcaceae, Lactobacillaceae, Leuconostocaceae, Clostridiaceae_1, Ruminococcaceae*, and *Peptostreptococcaceae* were dominant within the *Firmicutes* phylum, while the *Proteobacteria* phylum was made up of *Enterobacteriaceae* (Figure 1E). *Streptococcaceae* and *Lactobacillaceae* were the two major families in ileal contents with opposing changes in abundance as protein concentration decreased. Proportion of *Streptococcaceae* changed from 58.16% and 35.31% to 52.71% (*P* < 0.05) when protein concentration changed from 18% and 15% to 12%, respectively. In contrast, proportion of *Lactobacillaceae* reached a peak of 24.18% in the 15% CP group compared with 17.75% in the 12% CP group (*P* < 0.05) and 3.74% in the 18% CP group (*P* < 0.05). Other families such as *Clostridiaceae_1* and *Micrococcaceae* had similar tendencies with *Lactobacillaceae*, while the variation of *Enterobacteriaceae* and *Leuconostocaceae* resembled that of *Streptococcaceae*.

**Figure 2:**
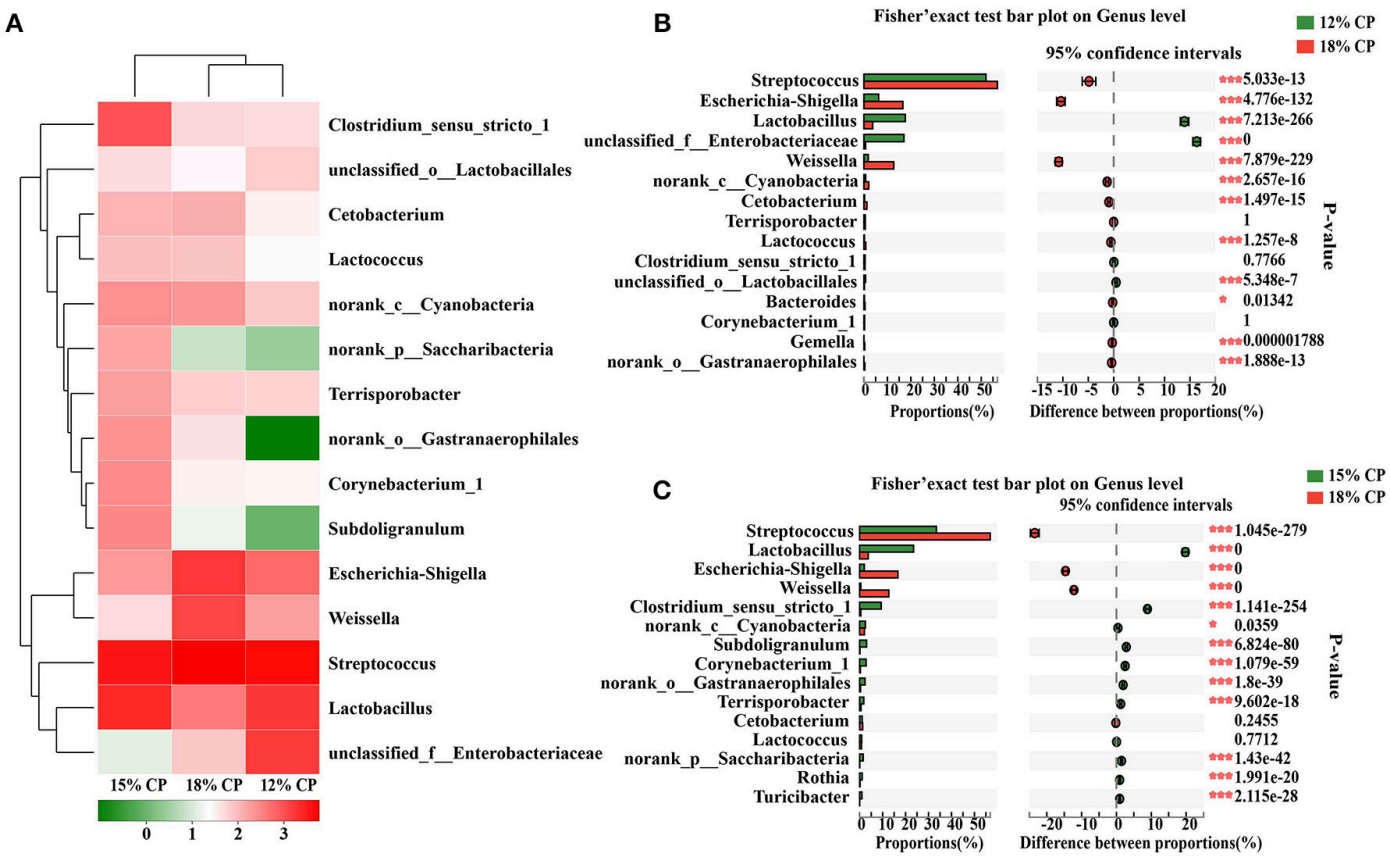
Effects of low-protein diets on the composition of ileal microbiota in growing pigs at the genus level. **(A)** Distribution of the top 15 ileal bacteria at the genus level. **(B)** Fisher' exact test bar plot on the genus level between the top 15 ileal bacteria of the 12 and 18% CP groups. **(C)** Fisher' exact test bar plot on the genus level between the top 15 ileal bacteria of the 15 and 18% CP groups.

At the genus levels, *Streptococcus, Lactobacillus, Escherichia-Shigella, Weissella*, and *Clostridium_sensu_stricto_1* were the predominant genera within *Streptococcaceae, Lactobacillaceae, Enterobacteriaceae, Leuconostocaceae*, and *Clostridiaceae_1* families, respectively (Figure 2A). Proportional variations of these genera were in accordance with their corresponding families in both 15% and 12% CP group with statistical significance (Figures 2B,C). In addition, several unique genera in the 15% CP group were rare compared to other groups such as *Rothia, Cetobacterium, Norank_p_Saccharibacteria*, and *Norank_o_Gastranaerophilalesand*, the appearance of these unique bacteria in the 15% CP-fed pigs should be evaluated further.

### Colonic bacterial community structure

**Figure 3:**
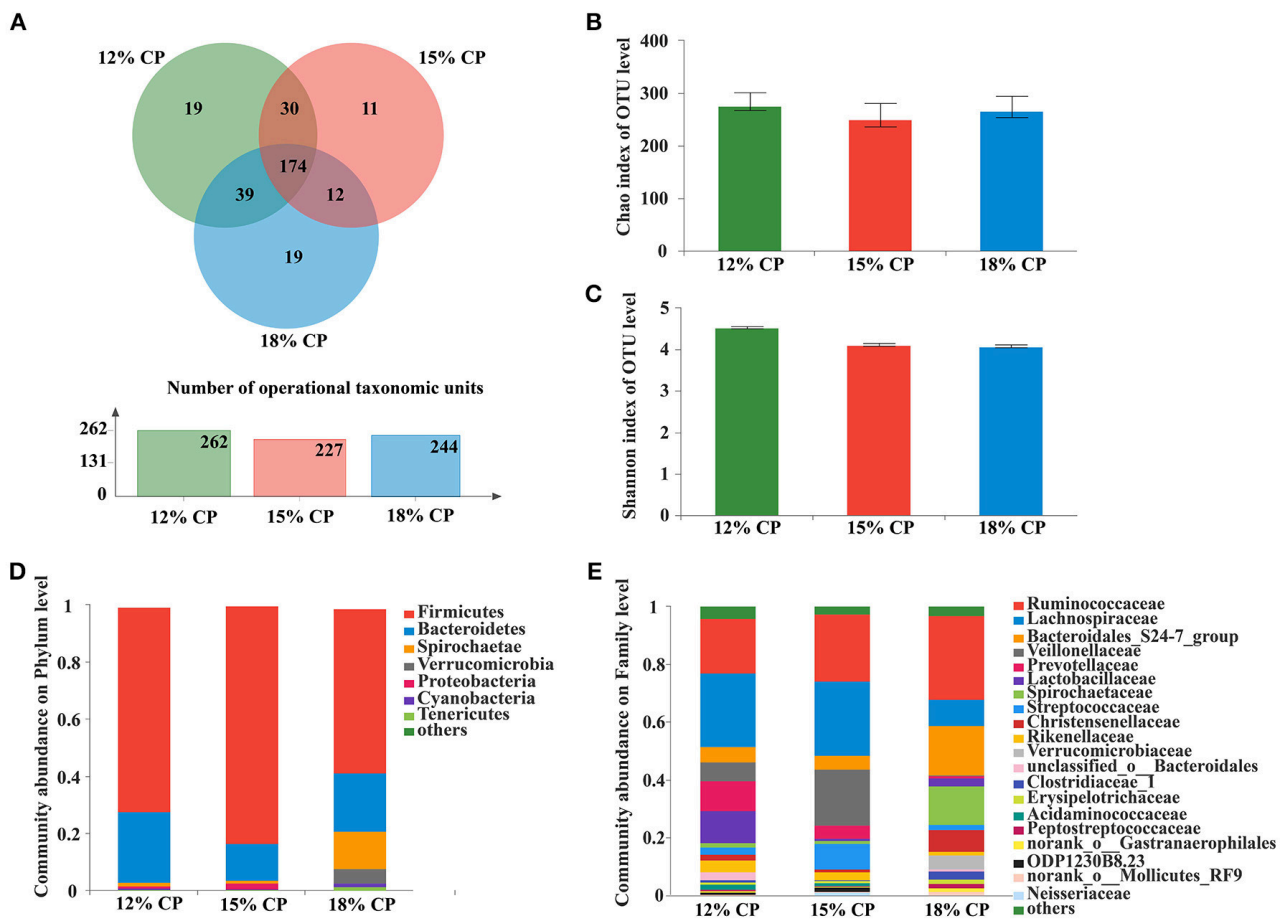
Effects of low-protein diets on the richness and composition of colonic microbiota in growing pigs at the phylum and family levels. **(A)** Venn of the operational taxonomic units (OTUs) among different dietary treatments in the colon. **(B)** Chao index of the bacterial community in the colon. **(C)** Shannon index of the bacterial community in the colon. **(D)** Distribution of colonic bacteria at the phylum level with relative abundance higher than 1%. **(E)** Distribution of colonic bacteria at the family level with relative abundance higher than 1%.

About 57.2% of the OTUs in the colonic bacterial community were shared among the three groups and each group contained similar amounts of unique OTUs (Figure 3A).

*Firmicutes, Bacteroidetes*, and *Spirochaetae* were the three major bacterial phyla in colonic contents and accounted for more than 90% of the total colonic bacterial community (Figure 3D). Their respective proportion altered with reduction of dietary protein concentration. When dietary protein concentration decreased to 15%, the proportion of *Firmicutes* reached a peak of 83.45%, while *Bacteroidetes* reached a minimum of 13.03%. With decreased protein concentration, the proportion of *Spirochaetae* plummeted from 13.39% in the 18% CP group to 1.36% in the 12% CP group. In addition, the proportion of *Verrucomicrobia* was essentially non-existent in the 12% and 15% CP groups.

At the family level, *Ruminococcaceae, Lachnospiraceae, Veillonellaceae, Lactobacillaceae*, and *Streptococcaceae* were dominant within the *Firmicutes* phylum in the colon. Proportion of *Lachnospiraceae* increased from 9.09% to 25.79% and 25.52% when dietary protein concentration decreased from 18% to 15% and 12%. Proportion of *Veillonellaceae* in the 15% CP group was 19.36%, which was higher than 18% CP group (Figure 3E). With decreasing dietary protein concentration, the proportion of *Ruminococcaceae* reduced from 28.99% in the 18% group to 23.11% and 18.90% in the 15% and 12% CP groups, respectively. *Bacteroidetes* phyla consisted mainly of *S24-7, Prevotellaceae*, and *Rikenellaceae*. When dietary protein concentration reduced from 18% to 12%, the proportion of *S24-7* decreased, while proportion of *Prevotellaceae* and *Rikenellaceae* gradually increased. *Spirochaetaceae* was the primary family within the *Spirochaetae* phylum and its proportion reduced sharply with the decrease of dietary protein concentration.

**Figure 4:**
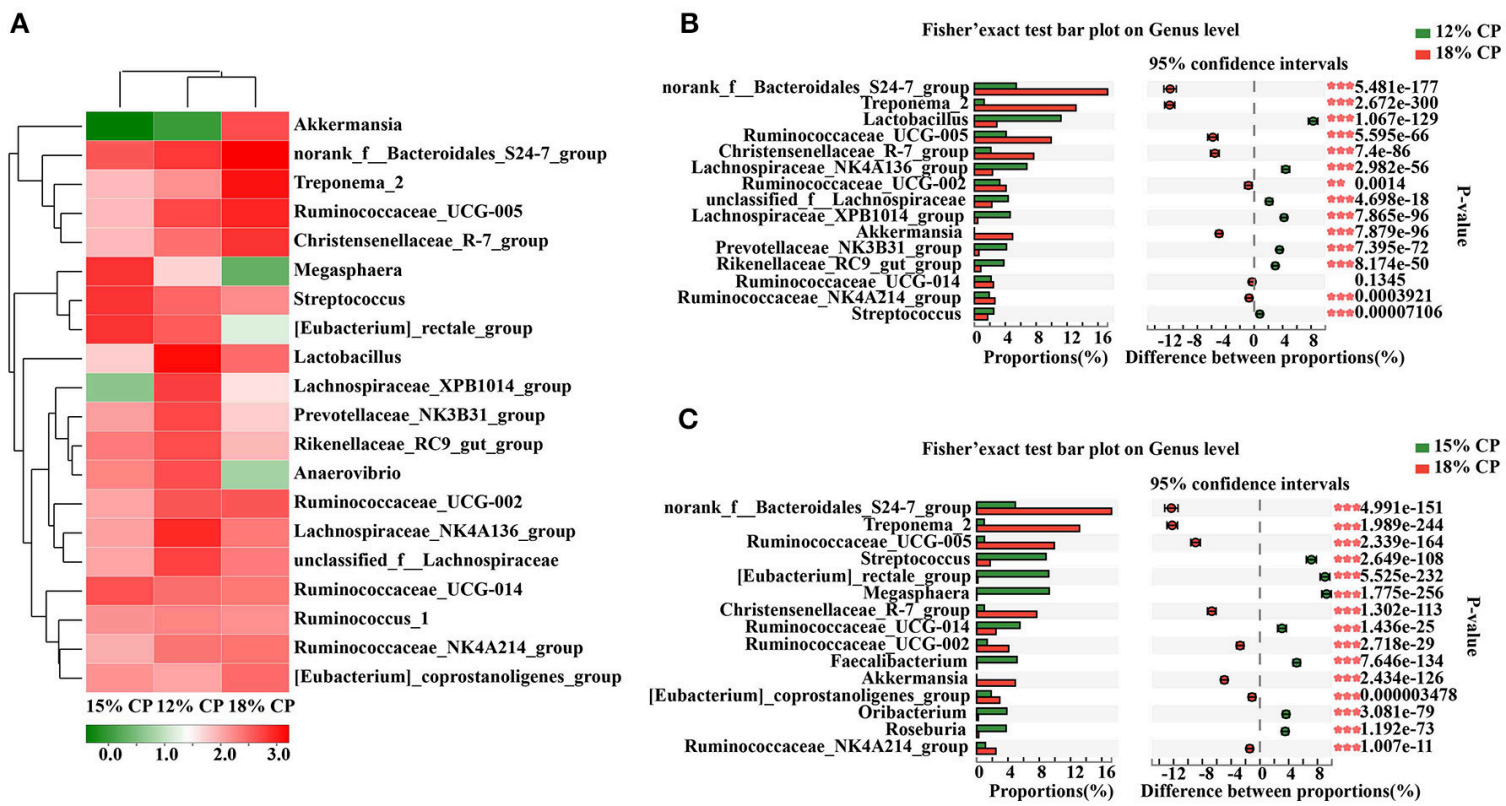
Effects of low-protein diets on the composition of colonic microbiota in growing pigs at the genus level. **(A)** Distribution of the top 20 colonic bacteria at the genus level. **(B)** Fisher' exact test bar plot on the genus level between the top 15 colonic bacteria of the 12 and 18% CP groups. **(C)** Fisher' exact test bar plot on the genus level between the top 15 colonic bacteria of the 15 and 18% CP groups.

At the genus level, *Ruminococcaceae_UCG-005, Lachnospiraceae_NK4A136_group, Lactobacillus*, and *Streptococcus* were the dominant genera of *Ruminococcaceae, Lachnospiraceae, Veillonellaceae, Lactobacillaceae*, and *Streptococcaceae*, respectively, whithin the *Firmicutes* phylum (Figure 4A). Proportion of *Ruminococcaceae_UCG-005* decreased with reduced protein concentration and was lowest at 0.98% in the 15% CP group (*P* < 0.05, Figure 4C). Proportion of *Lachnospiraceae_NK4A136_group* and *Lactobacillus* increased as CP decreased from 18% to 12% (*P* < 0.05, Figure 4B). In the family of *Bacteroidetes, Norank_f_Bacteroidales_S24-7_group, Prevotellaceae_NK3B31_group* and *Rikenellaceae_RC9_gut_group* were the dominant genera of *S24-7, Prevotellaceae*, and *Rikenellaceae* families, respectively. *Treponema_2* was dominant within the *Spirochaetae* family. The response to dietary protein concentration of most of these genera shared similar tendencies with their corresponding families (Figures 4B,C).

### Concentration of intestinal SCFAs

Concentrations of acetic acid in ileal contents tended to increase when dietary protein concentration decreased from 18% to 15%, but concentration of butyric acid and propionic acid did not differ among the three groups (Table 1). Isobutyric acid, isovaleric acid as well as valeric acid were presented in minimal concentration in the ileal contents. As for colonic contents, when dietary protein concentration decreased from 18% to 15%, concentration of acetic acid declined significantly (*P* < 0.05), but no notable difference in the concentration of propionic acid, isobutyric acid, butyric acid, isovaleric acid, and valeric acid was observed among the three groups.

**Table 1:** Effects of low-protein diets on short-chain fatty acids (SCFAs) concentration in intestinal contents of growing pigs.

**Items (mg/g)**	**Dietary protein concentration (%)**			***P*-value**
	**12**	**15**	**18**	
**ILEAL CONTENTS**				
Acetic acid	0.66 ± 0.17	1.20 ± 0.19	0.81 ± 0.01	>0.05
Propionic acid	0.49 ± 0.04	0.58 ± 0.02	0.57 ± 0.005	>0.05
Isobutyric acid	0.0011 ± 0.0002	0.0009 ± 0.0003	0.0011 ± 0.0003	>0.05
Butyric acid	0.12 ± 0.02	0.12 ± 0.01	0.17 ± 0.02	>0.05
Isovaleric acid	0.0067 ± 0.0001	0.0071 ± 0.0009	0.0072 ± 0.001	>0.05
Valeric acid	0.0083 ± 0.0009	0.0085 ± 0.0005	0.0086 ± 0.0007	>0.05
**COLONIC CONTENTS**				
Acetic acid	1.93 ± 0.23^b^	1.89 ± 0.16^b^	3.39 ± 0.52^a^	< 0.05
Propionic acid	1.16 ± 0.15	0.93 ± 0.08	1.60 ± 0.31	>0.05
Isobutyric acid	0.14 ± 0.04	0.12 ± 0.008	0.16 ± 0.05	>0.05
Butyric acid	0.83 ± 0.22	0.67 ± 0.05	1.15 ± 0.24	>0.05
Isovaleric acid	0.27 ± 0.07	0.24 ± 0.009	0.27 ± 0.09	>0.05
Valeric acid	0.22 ± 0.06	0.19 ± 0.02	0.26 ± 0.03	>0.05

a, b *Different superscript within a row means significantly different (P < 0.05). Values are means ± SEMs, n = 6*.

### Concentration of intestinal biogenic amines

When dietary protein concentration was reduced by 3 percentage points from 18% to 15%, concentrations of 5 biogenic amines were reduced slightly in both ileal and colonic contents without significance (Table 2). When dietary protein concentration reduced from 18% to 12%, the concentrations of putrescine, histamine, and spermidine in ileum and cadaverine and spermidine in colon decreased (*P* < 0.05).

**Table 2:** Effects of low-protein diets on biogenic amines concentration in intestinal contents of growing pigs.

**Items (μg/g)**	**Dietary protein concentration (%)**			***P-*value**
	**12**	**15**	**18**	
**ILEAL CONTENTS**				
Methylamine	8.33 ± 1.07	11.8 ± 0.50	11.5 ± 1.38	>0.05
Cadaverine	22.2 ± 2.81	30.6 ± 2.74	31.1 ± 5.43	>0.05
Putrescine	31.3 ± 1.23^b^	42.0 ± 3.47^ab^	48.3 ± 4.53^a^	< 0.05
Histamine	41.3 ± 2.07^b^	54.5 ± 1.03^a^	58.5 ± 2.25^a^	< 0.05
Spermidine	40.7 ± 1.41^b^	49.6 ± 2.56^ab^	53.5 ± 2.44^a^	< 0.05
**COLONIC CONTENTS**				
Methylamine	13.8 ± 2.18	20.0 ± 4.09	22.2 ± 4.28	>0.05
Cadaverine	32.4 ± 1.51^b^	42.8 ± 2.99^a^	43.3 ± 2.28^a^	< 0.05
Putrescine	44.8 ± 1.64	57.5 ± 7.26	61.0 ± 3.48	>0.05
Histamine	64.3 ± 3.11	73.1 ± 5.31	80.1 ± 4.88	>0.05
Spermidine	23.1 ± 4.44^b^	30.4 ± 3.71^ab^	41.2 ± 2.58^a^	< 0.05

a, b *Different superscript within a row means significantly different (P < 0.05). Values are means ± SEMs, n = 6*.

### Intestinal morphology and permeability

**Figure 5:**
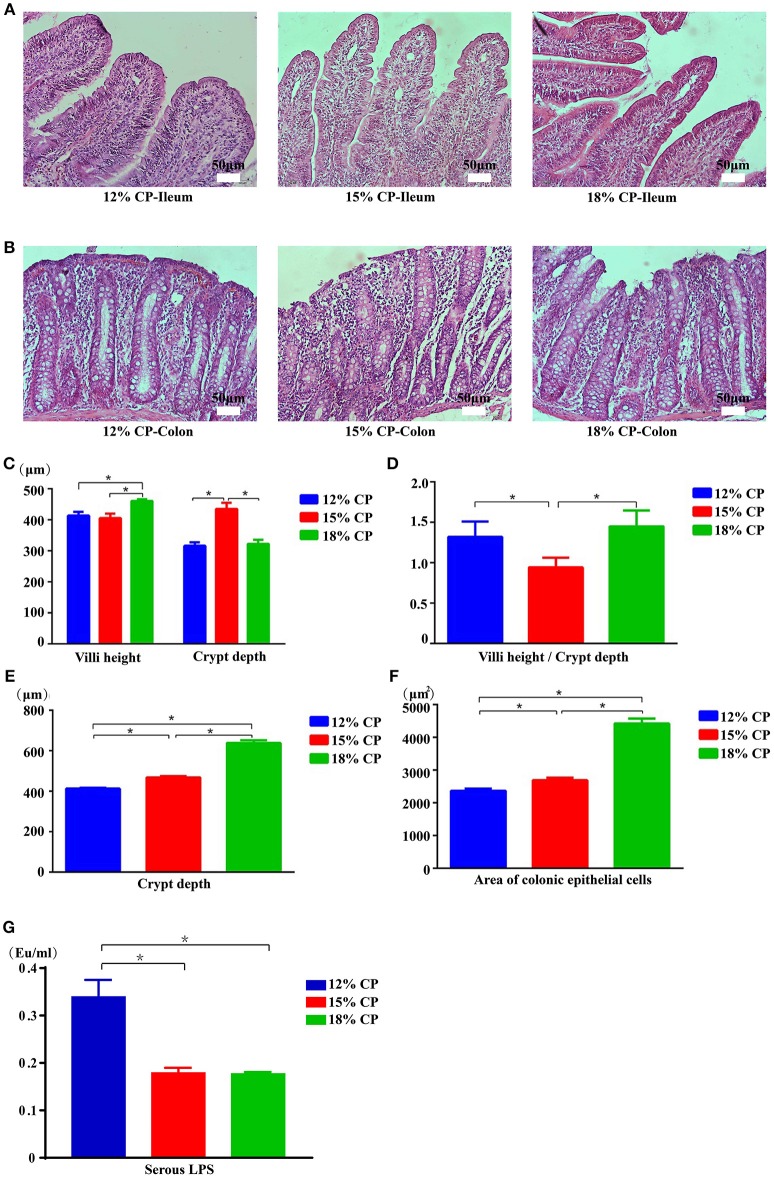
Effects of low-protein diets on intestinal mucosal morphology and permeability of growing pigs (the stained sections were photographed at 200 × magnification). **(A)** Ileal morphology observation of different dietary protein groups. **(B)** Colonic morphology observation of different dietary protein groups. **(C)** Villi height and crypt depth of the ileum. **(D)** Villi height to crypt depth ratios in the ileum. **(E)** Colonic crypt depth. **(F)** Area of colonic epithelial cells. **(G)** Lipopolysaccharide (LPS) level in the serum. Values are means ± standard deviations (*n* = 6). Star indicates a significant difference (*P* < 0.05).

Effects of low-protein diets on the intestinal morphology of growing pigs were determined (Figures 5A,B). Ileal morphology in the 18% CP group was more integrated than those in the 15% and 12% CP groups with fewer intestinal villi falling away, but no apparent damage was observed in the colon among the different groups. In the ileum, villi height in the 18% CP group was highest and crypt depth in the 15% CP group was highest (*P* < 0.05, Figures 5C,D). For colonic tissue, both crypt depth and area of colonic epithelial cells gradually reduced with decreasing dietary protein concentration (*P* < 0.05, Figures 5E,F). Effects of low-protein diets on intestinal permeability were detected with serous LPS level (Figure 5G). However, there was no significant change among the three groups.

### Expression of intestinal tight junction proteins and biomarkers of ISCs

**Figure 6:**
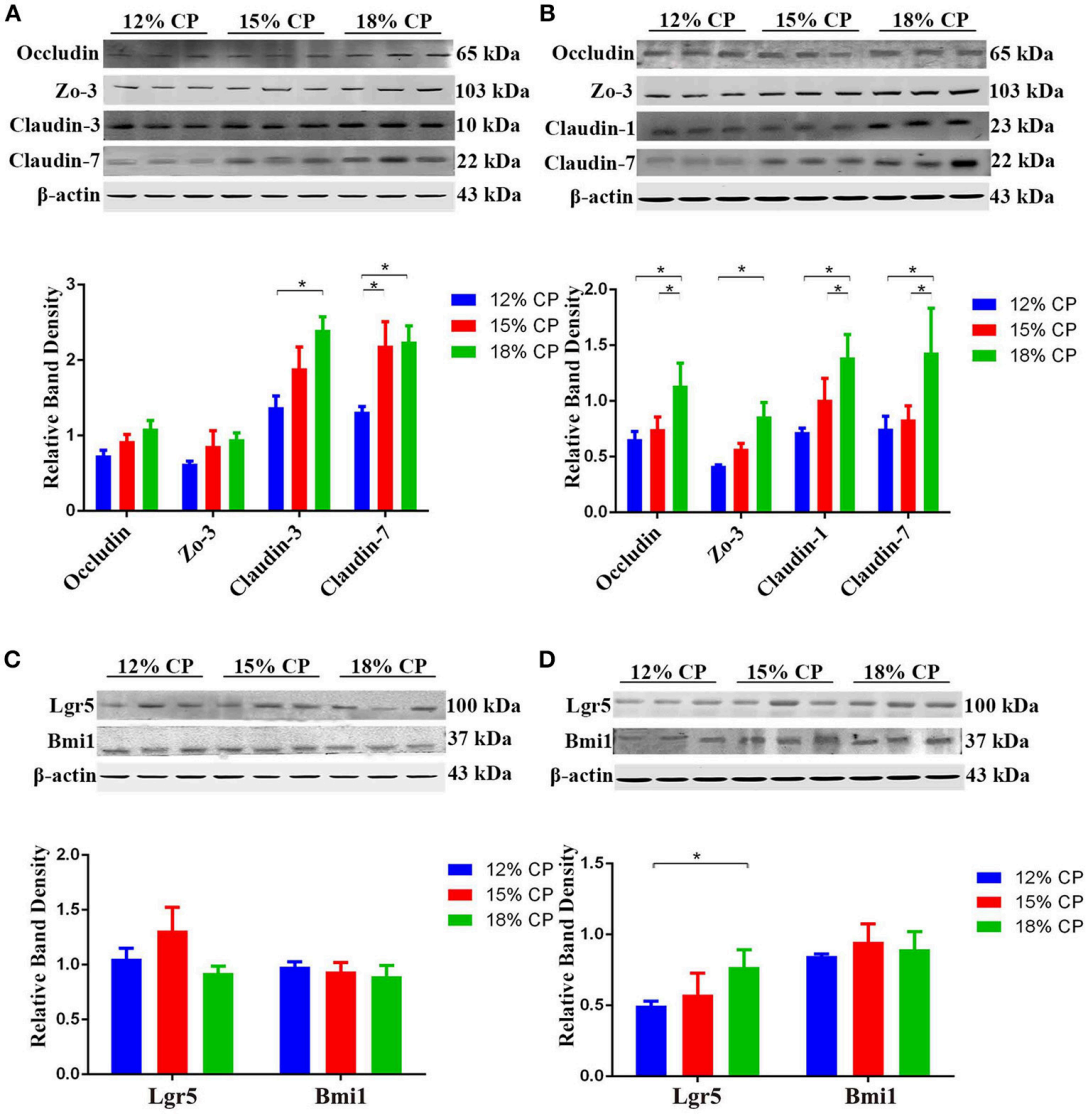
Effects of low-protein diets on the expression of intestinal tight junction proteins and biomarkers of intestinal stem cells of growing pigs. **(A)** Expression of occludin, ZO-3, claudin-3, and claudin-7 in ileal tissue. **(B)** Expression of occludin, ZO-3, claudin-1, and claudin-7 in colonic tissue. **(C)** Expression of Lgr5 and Bmi1 in ileal tissue. **(D)** Expression of Lgr5 and Bmi1 in colonic tissue. Values are means ± standard deviations (*n* = 3). Star indicates a significant difference (*P* < 0.05).

Effects of low-protein diets on expression of intestinal tight junction proteins and biomarkers of ISCs were evaluated. In the ileum, expression of claudin-3 decreased significantly as dietary protein concentration reduced from 18% to 12% and expression of claudin-7 in the 12% CP group was lower than other two diet groups (*P* < 0.05, Figure 6A). In the colon, compared to the 18% CP group, occludin, claudin-1 and claudin-7 decreased significantly in the 15% CP group and occludin, ZO-3, claudin-1, and claudin-7 declined dramatically in the 12% CP group (*P* < 0.05, Figure 6B). Lgr5 and Bmi1 are biomarkers of ISCs. The expression of Lgr5 in colon decreased significantly from 18% to 12% CP group (*P* < 0.05, Figure 6D). Remarkably, ileal expression of Lgr5 in the 15% CP group increased slightly compared to the other two groups (Figure 6C).

### Immunofluorescence assay

**Figure 7:**
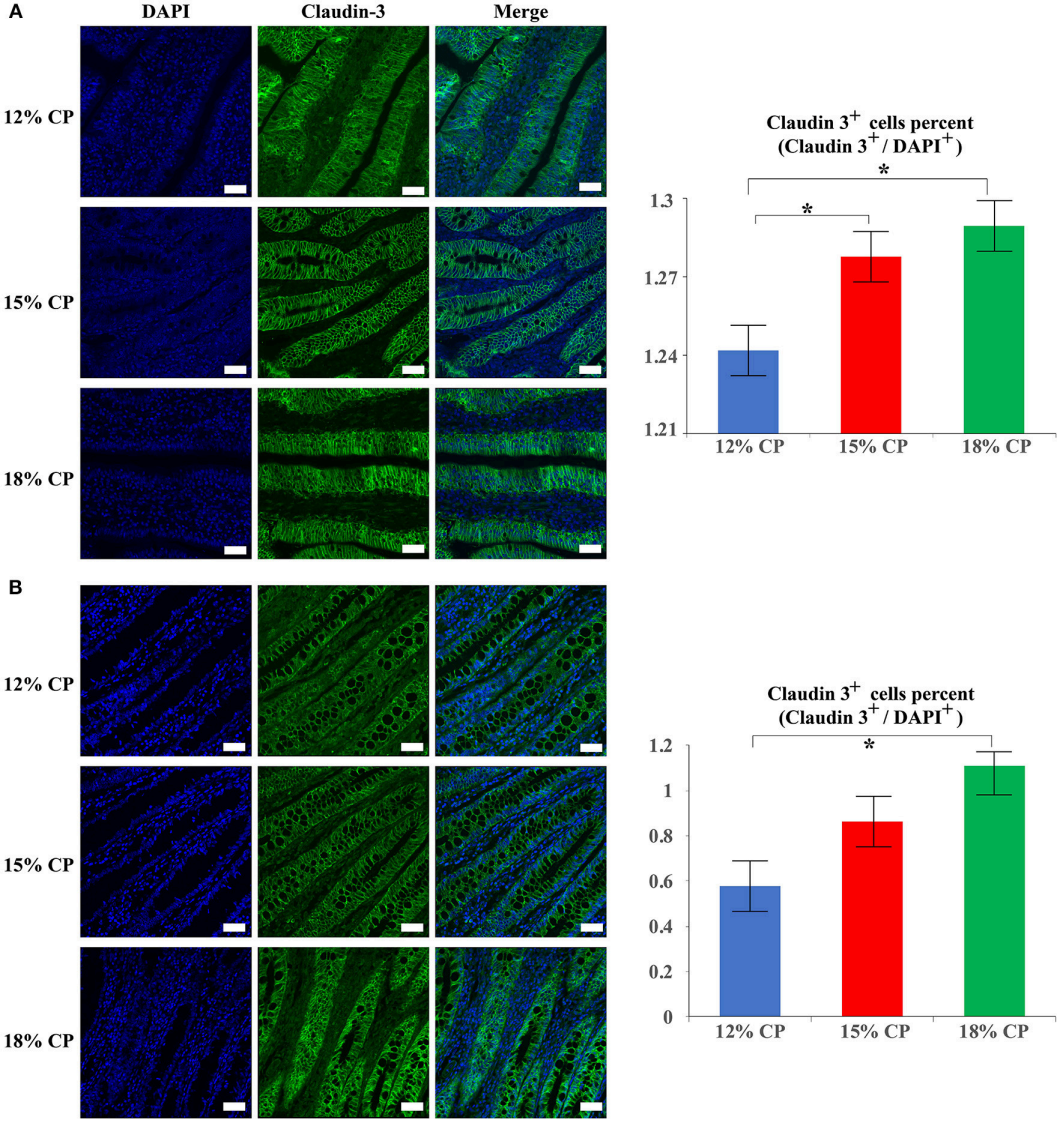
Effects of low-protein diets on staining of claudin-3 in the ileum and colon in growing pigs. **(A)** Distribution and intensity of claudin-3 staining in ileal tissue. **(B)** Distribution and intensity claudin-3 in colonic tissue. Values are means ± standard deviations (*n* = 3). Scale bar: 20 μm. Star indicates a significant difference (*P* < 0.05).

In addition to protein expression, the distribution of tight junction proteins is also important for the formation of tight junction. Thus, the distribution of claudin-3 in the ileum and colon were detected. In the 18% and 12% CP group, ileal claudin-3 was distributed all over the cytoplasm, while claudin-3 in the 15% CP group was mainly located in cell membrane (Figure 7A). In the colon, the outline of colonic epithelial cells in the 15% CP group were more clearly visible than the other two groups, but not as significant as that in the ileum (Figure 7B).

## Discussion

A low-protein diet is crucial for addressing environmental problems and saving protein resources, but its influence on the gut micro-environment is still not fully understood. This study aimed at investigating the effect of dietary protein on intestinal microbiota and functions.

In the present study, low-protein diets altered the intestinal bacterial community of growing pigs. Chao estimates indicated the bacterial richness and Shannon index reflected the bacterial diversity. The highest richness and diversity of ileal bacteria in the 15% CP group suggested that moderate protein deficiency may promote the growth of ileal bacteria, which was consistent with Fan et al. (2017a). The lower Shannon index in the ileum of the 12% CP group indicated that excessive protein deficiency may inhibit the diversity of intestinal bacteria. In the colon, both bacterial richness and diversity were only slightly affected by reducing protein concentration, which may be related to the high stability of colonic bacterial populations. Therefore, a 3-percentage reduction of the level of dietary protein concentration was possible to adjust the gut microbiota in ileum.

As for the ileal microbiota, when dietary protein dropped by 3 percentage points, the decreased *Enterobacteriaceae* within the *Proteobacteria* phyla has been considered to contain many pathogenic bacteria (Lv et al., 2016), indicating the potential for inhibiting pathogens with moderate dietary protein restriction. The decline of *Streptococcus* and *Escherichia-Shigella* in the ileum when dietary protein concentration decreased by 3 percentage points suggested the positive effect. According to a previous study, *Streptococcus* and *Escherichia-Shigella* were involved in amino acid utilization (Neis et al., 2015). Thus, the drop of *Streptococcus* and *Escherichia-Shigella* could be explained by the shortage of protein substrate for fermentation, which is consistent with previous studies (Zhou et al., 2016; Fan et al., 2017a). *Streptococcus* and *Escherichia-Shigella* can cause numerous infections and diseases, such as scarlet fever and bacillary dysentery (Pop et al., 2014; Davies et al., 2015). Bacteria belonging to *Escherichia-Shigella* were also reported to be associated with necrotizing enterocolitis and inhibition of intestinal peristalsis (Brower-Sinning et al., 2014). The low density of *Escherichia-Shigella* in the 15% CP group along with the high concentration of acetic acid may indicate a preference for weakly alkaline environments by *Escherichia-Shigella*; a reduction of these bacteria in the presence of an acidic environment was also reported in another study (Fan et al., 2017a). *Lactobacillus, Clostridium_sensu_stricto_1, Bifidobacterium*, and *Rothia* in ileal contents increased in the 15% CP group compared with 18% CP group. *Lactobacillus* is one of the predominant genera in the ileum of growing pigs. *Lactobacillus* and *Bifidobacterium* can ferment carbohydrates into lactic acid and improve the intestinal environment (Kleerebezem et al., 2003; Fukuda et al., 2011). *Rothia* can produce butyric acid. *Clostridium_sensu_stricto_1* is reported to protect against colonization of bacterial pathogens (Kim et al., 2017). Thus, by strengthening beneficial microbial populations and suppressing harmful bacterial growth in the ileum, moderate protein restriction can optimize the microbiota structure. However, the decrease of beneficial microbiota such as *Leuconostocaceae* in the 12% CP group was likely due to the injury of ileal barrier function. Thus, diets with moderate protein restriction can optimize ileal microbial structure but a dramatic decline in dietary protein may weaken host resistance to certain intestinal diseases.

In the colon, when dietary protein concentration declined, the abundance of *Firmicutes* increased while *Bacteroidetes, Spirochaetae*, and *Verrucomicrobia* decreased. The ratio of *Firmicutes* to *Bacteroidetes* has been reported to increase in obese animals (Bäckhed et al., 2004) and is related to energy intake of the host (Cottenie, 2005; Ley et al., 2006). In this study, the increasing ratio was consistent with the research on low protein diets and their role in increasing carcass fat deposition (Fan et al., 2017a). The colonic abundance of *Norank_f_Bacteroidales_S24-7_group, Treponema*_*2*, and *Ruminococcaceae_UCG-005* decreased with reduced protein concentration. Altered abundance of *S24-7* is associated with diet composition and is abundant in diabetes-sensitive mice induced by high-fat diet (Ormerod et al., 2016). Thus, the decreasing *S24-7* family and protein-degrading *Ruminococcaceae* reflected a reduced metabolic activity in the presence of host dietary deficiency. Lange reported that consuming potato starch chronically benefits health and reduces the abundance of *Treponema* (Lange et al., 2015). Corn comprises a large proportion of low-protein diet formulations so there will be elevated resistant starch entering the colon for microbial metabolism. *Lachnospiraceae*, which is saccharolytic and can degrade cellulose, was elevated in the low protein group of this research. *Treponema*_*2* is also considered a disease-causing bacterium, while the *S24-7* family is differentiated by their degree of IgA- labeling (Palm et al., 2014; Bunker et al., 2015) which indicates that the *S24-7* family are related with the innate immune system. *Prevotellaceae* is reported to relate to several diseases, such as asthmatic airway inflammation, autism, and arthritis (Biddle et al., 2013; Scher et al., 2013; Clarke et al., 2014; Lukens et al., 2014). Alteration of microbiota especially increasing *Prevotellaceae* is a key factor in regulating inflammation and caspase-8 mediated IL-1β maturity (Lukens et al., 2014) and associated mucin degradation resulting in reduced layer of mucin on the intestinal epithelium (Brinkman et al., 2011). *Veillonellaceae* decreased with reducing protein concentration and could transform acids produced by other bacteria into more acidic products. Thus, there were no specific orientation in the change of colonic microbiota. Broadly, protein deficiency may disturb homeostasis of colonic microbiota involving metabolism and immunity and further affect the host.

SCFAs, mainly composed of acetate, propionate and butyrate, are produced by fermentation of undigested dietary proteins and fibers. Branched chain fatty acids (BCFAs), which refer to isobutyrate and isovalerate, are produced by the deamination of branched chain amino acids such as leucine and valine by bacteria (Lv et al., 2016). Functionally, SCFAs have profound effects on metabolism and gut health (Tan et al., 2014). Acetate and propionate are energy substrates for peripheral tissues and butyrate is preferentially used as an energy source by colonic epithelial cells (Tremaroli and Bäckhed, 2012). As SCFA can stimulate the growth of the mucosa, the rise of ileal acetate in the 15% CP group was consistent with optimization of the microbial structure. Most SCFAs in colon decreased by low-protein diets, suggesting a weaker bacterial fermentation in colon caused by nitrogen reduction. The lower concentration of SCFAs in the colon of 12% CP group may contribute to disorders in colonic microbiota without suppressing the proliferation of pathogenic microorganism. Therefore, moderate protein restriction by 3 percentage enhanced the ileal microbial fermentation and SCFA formation, while reducing protein concentration by 6 percentages may inhibit the bacterial fermentation.

Another product of microbial metabolism, biogenic amines, decreased with the reduced dietary protein in both the ileum and colon, which was consistent with other studies (Fan et al., 2017b), indicating protein fermentation in the intestine decreased. Since putrescine, spermidine, and cadaverine can protect intestinal mucosa and epithelial cells as reported (Sasaki et al., 2001; Penrose et al., 2013), their sharp decrease may explain the injury of intestinal morphology.

Reduction of dietary protein concentration by 3–6% points damaged both the ileal and colonic mucosal morphology resulting in lower villi height and higher crypt depth as well as decreased area of colonic epithelial cell in present study, which indicated the insufficient development both in ileum and colon. Different sections of the digest tract assume different responsibilities. Since the intestinal surface area represented by the tight packing and long projections of villi showed the maximal absorption of nutrients allowed (Shyer et al., 2015), the decreased ratio of villi height to crypt depth in the ileum of the 15% CP group showed reduced nutrient absorption. Crypts are places for synthesis of ISCs and transit amplifying cells, with crypt depth reflecting the proliferation of intestinal epithelial cells. In our research, the increased crypt depth in the ileum of the 15% CP group showed the potential for intestinal epithelial proliferation, while the colonic epithelial proliferation decreased with protein restriction. Lots of bacteria and LPS exists in the lumen of mammals. In case of the injured intestinal barrier, LPS go through the intestinal mucosa and enter blood circulation, prior to bacteria with lower molecular weight (Haller et al., 2004; Arrieta et al., 2009). Thus, serous LPS level can also characterize the function of intestinal permeability. The stable level of LPS in the 15% CP group and increased serous LPS in the 12% CP group indicated moderate protein restriction maintain the intestinal barrier function while 6% of protein restriction did harm to intestinal functions.

The intestinal barrier is regulated by a well-organized system known as the tight junction, which consists of several unique proteins including the junction adhesion molecule, the trans-membrane protein occludin, and members of the claudin family as well as linker proteins such as the zonula occludens protein family (Peterson and Artis, 2014). The tight junctions are intercellular protein located at the apical portions of the membranes of intestinal epithelial cells (Marchiando et al., 2010). Morphological alteration of the tight junctions both in protein expression and distribution may cause intestinal disorder (Suzuki, 2013). Claudins have strong adhesion functionand can form intercellular connections and block the intercellular space (Ciana et al., 2010). It has been reported claudin molecules, not occludin, were polymerized within the membranes to reconstitute paired tight junctions and were major structural components of tight junctions (Kiuchi-Saishin et al., 2002). In consideration of a healthier pattern of ileal bacterial community in the 15% CP group, ileal expression of important intestinal tight junction proteins had no significant reduction, while ileal distribution of claudin-3 shifted from the cytoplasm to the cell membrane in comparison with that in the 18% CP group, indicating the complete morphology of the ileal tight junctions. When the margin of decrease reached 6 percentage points, claudin-3, and claudin-7 decreased their expression significantly in both ileum and colon. In accordance with disorders of colonic microbiota and absorption, intestinal barrier function of the colon, reflected by occludin, claudin-1, claudin-7, and ZO-3 protein expression as well as Lgr5 for ISC, gradually reduced with decreasing protein concentration. In our study, moderate reduction of protein concentration altered the distribution of claudin-3 in the ileum, which agrees with the optimization of intestinal flora. And both of the factors may compensate for moderate protein reduction, while it cannot compensate for 6 percent protein reduction.

The intestinal epithelial barrier is initiated with the ISC niche, which develops the differentiated cell types including Paneth cells, goblet cells, enteroendocrine cells, and absorptive enterocytes or colonocytes (Peterson and Artis, 2014). Lgr5 and Bmi1 are biomarkers of CBC ISCs (the crypt base columnar stem cell) and “+4” ISCs. CBC ISCs at the base of crypt are active and respond to stimulation, while “+4” ISCs above the Paneth cell are usually stable and only activated in stressed state to compensate for the loss of ISC (Tian et al., 2011, 2015; Philpott and Winton, 2014). In this study, the expression of Bmi1 was stable with decreasing dietary protein, indicating no severe injury in the ileum and colon. The increase of Lgr5 in the 15% CP group indicated the potential for the proliferation of ISCs marked by Lgr5 in the ileum. Thus, microbiota in the 15% CP group may have stimulated CBC ISCs and improved ileal barrier function, and is supported by the increased *Lactobacillus* which have been reported to stimulate gut epithelial proliferation via Nox-mediated generation of reactive oxygen species (Jones et al., 2013).

However, previous study using an adult pig model reported a different impact of reduced dietary protein on pig growth, intestinal health and microbiota. For example, in adult pigs, at a similar level of dietary protein restriction (3% lower than control group), the growth performance and meat quality were impaired (Becker and Yu, 2013), and *Peptostreptococcaceae* and the ileal barrier function were reported to increase (Fan et al., 2017a). It is expected that the variation in response dietary protein restriction is due to differences in nutritional needs where protein and calcium are essential for lean tissue deposition which is the primary tissue of growth in growing animals. However, lipid deposition, and hence energy and dietary lipid content, contribute substantially to nutritional needs of adult pigs (Hollister et al., 2014). In conclusion, reduction of dietary protein concentration by 3 percentage points could optimize ileal microbiota, with decreasing *Streptococcus*, increasing *Lactobacillus* and *Bifidobacterium*, and improved proliferation of intestinal stem cell as well as alteration of tight junction protein distribution without affecting intestinal barrier function. However, 6 percentage points of reduction in dietary protein affected the bacterial community structure in both ileum and colon of growing pigs and weakened the colonic tight junction function. The modified microbiota structure in the ileum of the 15% CP group can improve CBC ISCs and strengthen intestinal barrier function.

## Author contributions

The research was mainly conceived and designed by XM, and conducted by XC, PS, and PF. XC and PF analyzed the data. XC wrote the manuscript. TH, CL, LeeJ, DJ, LinJ, NM, YC, JieZ, LinJ, JinZ, and XM critically reviewed the manuscript. XC and XM had primary responsibility for final content. All authors read and approved the final manuscript.

### Conflict of interest statement

The authors declare that the research was conducted in the absence of any commercial or financial relationships that could be construed as a potential conflict of interest.

## Abbreviations

Bmi1, proto-oncogene: polycomb ring finger
BW: body weight
CBC: the crypt base columnar stem cell
CP: crude protein
DE: digestible energy
ISC: intestinal stem cell
Lgr5: leucine rich repeat containing G-protein-coupled receptor 5
LPS: lipopolysaccharide
NRC: National Research Council
OTU: operational taxonomic unit
SCFA: short-chain fatty acid
SID: standardized ileal digestible
VWD: variable wavelength detector
ZO-3: zonula occludens protein 3.
