# Supplementary material for: Moderate Dietary Protein Restriction Optimized Gut Microbiota and Mucosal Barrier in Growing Pig Model
Source: Front Cell Infect Microbiol. 2018 Jul 18;8:246. doi: 10.3389/fcimb.2018.00246 (PMC6058046; doi:10.3389/fcimb.2018.00246)
Supplement: Supplementary file 1 [file Table_1.DOCX]

**Supplemental Table 1** The composition and nutrient content of diets (%, as-fed basis) ^a^.

| Items | Diets | | |
| --- | --- | --- | --- |
|  | 12% CP | 15% CP | 18% CP |
| Ingredients ^b^ |  |  |  |
| Corn | 77.60 | 67.50 | 58.60 |
| Soybean meal | 10.00 | 19.50 | 29.00 |
| Wheat bran | 5.06 | 6.94 | 7.80 |
| Soybean oil | 3.00 | 2.38 | 1.55 |
| *L*-Lysine HCl, 98.5% | 0.74 | 0.46 | 0.18 |
| *DL*-Methionine, 98.0% | 0.17 | 0.09 | 0.00 |
| *L*-Threonine, 98.0% | 0.26 | 0.14 | 0.01 |
| *L*-Tryptophan, 98.0% | 0.07 | 0.02 | 0.00 |
| Dicalcium phosphate | 0.90 | 0.78 | 0.69 |
| Limestone | 0.90 | 0.89 | 0.87 |
| Salt | 0.30 | 0.30 | 0.30 |
| Premix ^c^ | 1.00 | 1.00 | 1.00 |
| Total | 100.00 | 100.00 | 100.00 |
| Nutrient levels ^d^ |  |  |  |
| Digestible energy (MJ/kg) | 14.20 | 14.20 | 14.20 |
| CP | 12.35 | 15.16 | 18.27 |
| SID Lysine | 0.94 | 0.97 | 0.97 |
| SID Methionine + Cysteine | 0.55 | 0.56 | 0.57 |
| SID Threonine | 0.60 | 0.61 | 0.61 |
| SID Tryptophan | 0.17 | 0.17 | 0.17 |
| SID Arginine | 0.57 | 0.82 | 1.08 |
| SID Histidine | 0.25 | 0.33 | 0.41 |
| SID Isoleucine | 0.35 | 0.49 | 0.64 |
| SID Leucine | 0.94 | 1.14 | 1.35 |
| SID Phenylalanine | 0.46 | 0.62 | 0.77 |
| SID Valine | 0.44 | 0.56 | 0.66 |
| Calcium | 0.61 | 0.63 | 0.60 |
| Total phosphorus | 0.45 | 0.48 | 0.51 |
| Essential AA | 5.03 | 6.04 | 7.00 |
| Non-essential AA | 5.83 | 7.50 | 9.12 |
| Essential AA/Non-essential AA | 0.86 | 0.81 | 0.76 |

^a^ CP, crude protein; SID, standard ileal digestible.

^b^ *L*-Lysine, *DL*-Methionine, *L*-Threonine, and *L*-Tryptophan were provided by Health & Nutrition of Evonik Industries AG, Germany.

^c^ Provied per kilogram of complete diet: vitamin A, 3,800 IU; vitamin D_3_, 800 IU; vitamin E, 9 mg; vitamin K_3_, 1 mg; vitamin B_2_, 2 mg; vitamin B_6_, 1.2mg; vitamin B_12_, 10 μg; niacin, 10 mg; biotin, 50 μg; folate, 0.4 mg; Mn, 3 mg; Zn, 80 mg; Fe, 80 mg; Cu, 5 mg; Se, 0.25 mg; I, 0.14 mg.

^d^ All nutrient levels except digestible energy were analyzed.
